# The association between smoking and clinical outcomes among spondylodesis patients: A systematic review and meta-analysis

**DOI:** 10.1371/journal.pone.0337799

**Published:** 2026-01-13

**Authors:** Mahnoor Shafi, Anna Baukje Lebouille-Veldman, Chady Omara, Namita Kaler, John Kilgallon, Sulaiman Sajed, Linda S. Aglio, Rania A. Mekary, Carmen Vleggeert-Lankamp

**Affiliations:** 1 Department of Neurosurgery, Computational Neuroscience Outcomes Center, Harvard Medical School, Brigham and Women’s Hospital, Boston, Massachusetts, United States of America; 2 Department of Neurosurgery, Johns Hopkins University School of Medicine, Baltimore, Maryland, United States of America; 3 Department of Neurosurgery, Leiden University Medical Center, Leiden, The Netherlands; 4 School of Pharmacy, Massachusetts College of Pharmacy and Health Sciences University, Boston, Massachusetts, United States of America; 5 Department of Anesthesiology, Perioperative and Pain Medicine, Brigham and Women’s Hospital, Boston, Massachusetts, United States of America; 6 Department of Neurosurgery, Spaarne Hospital, Haarlem/Hoofddorp, The Netherlands; Korea University College of Medicine / Korea University Guru Hospital, KOREA, REPUBLIC OF

## Abstract

**Background:**

The effects of smoking on outcomes after spinal spondylodesis remain unclear due to conflicting findings in the existing literature. This meta-analysis aimed to evaluate the association between smoking and clinical as well as radiological outcomes following instrumented spinal fusion, and to examine differences based on smoking status, spinal region, and surgical extent.

**Methods:**

PubMed, Embase, and Cochrane were searched up to August 2024 for comparative studies reporting outcomes in smokers and non-smokers undergoing spinal fusion. Extracted outcomes included clinical scores (Oswestry Disability Index [ODI], Neck Disability Index [NDI], Visual Analog Scale [VAS] for pain), fusion rates, pseudoarthrosis, and complications. Pooled percent mean change from baseline and weighted incidences with 95% confidence intervals (CI) were calculated using a random-effects model. Subgroup analyses were performed by spinal region, number of operated levels, and smoking history (current, former, never).

**Results:**

Twenty-nine studies involving 6,687 patients were included. In most outcome measures, smokers showed smaller percent improvements than non-smokers. For instance, NDI improved by 42.8% (95% CI: 25.4–60.1%) in smokers vs. 49.7% (32.4–64.0%) in non-smokers; ODI improved by 48.6% (34.7–62.5%) vs. 56.5% (42.6–70.3%); and VAS back pain by 55.0% (29.7–80.2%) vs. 60.5% (35.2–85.7%). Fusion rates were lower in smokers (86.8%) than in non-smokers (95.1%), while pseudoarthrosis was more common in smokers (17.2% vs. 7.3%). Subgroup analyses revealed similar trends across spinal regions and surgical scope. A hierarchical pattern was observed, with never-smokers experiencing the most favorable outcomes, followed by former smokers.

**Conclusion:**

In most instances, smokers appeared to experience worse outcomes following instrumented spinal procedures compared to non-smokers, and a hierarchical pattern was notable, with current smokers experiencing the worst outcomes, followed by former smokers compared to never-smokers. Future well-designed studies with proper adjustment for confounding are needed to further confirm these findings.

## Introduction

### Background

Despite the decline of cigarette smoking over the last 25 years, smoking still remains a health concern to health-care providers all over the world [1]. Smoking continues to be the primary cause of morbidity and mortality causing around one in five deaths each year in the United States [2]. In spine surgery, the negative effects of smoking have been reported several times with respect to bone healing. The following processes play an explanatory role: nicotine is responsible for inhibiting tumor necrosis factor alpha (a cytokine that is crucial to the inflammatory response), angiogenesis, and osteogenesis, processes crucial to bone metabolism; dioxin, is postulated to adversely affect osteogenesis; and polycyclic aryl hydrocarbon compounds bind osteoblast and osteoclast receptors causing significant bone metabolism impairment [3–6].

The negative effects of smoking, however, are not confined to bone health. In fact, research has demonstrated that tobacco not only negatively influences vitamin D and calcium absorption, but also established negative effects on parathyroid hormones, adrenal hormones, gonadal hormones and oxidative stress. All these factors have a detrimental effect on the body’s hemostasis. This suggests that the effect of smoking in relation to spinal surgery is not exclusive to bone healing, radiological outcomes, and surgical site infections; effects that are oftentimes the primary focus of spine surgery research [7–12] but is also a key player in the whole body’s metabolism and thus plays an overall crucial role in physical health and clinical outcomes after surgery. Definitive reports of the direct influence of smoking around the time of surgery vary with some studies observing a negative outcome postoperatively due to preoperative smoking, while other original studies found no measurable influence on the functional outcome of patients following spinal spondylodesis. [13,14].

Although smoking is associated with a high rate of reoperation, current guidelines for spondylodesis do not restrict patients from smoking prior to surgery [15]. At this moment, it is still difficult to answer the question of whether or not a patient will significantly benefit from stopping smoking following instrumented spinal surgery with regard to clinical outcomes. Thus, concrete and cumulative evidence is required to emphasize the risk of smoking on patient outcomes and the possible benefits of smoking cessation prior to spinal fusion surgery.

A previously conducted systematic review suggested that radiological fusion may also depict the effects of smoking on patients undergoing spondylodesis, with findings such as pseudoarthrosis and non-union [16]. However, this review and other meta-analyses on this subject focused on the effects of smoking on radiological parameters [16–19]. While a limited number of systematic reviews [20,21] and meta-analyses [16–19] have investigated the relationship between smoking and fusion, these have largely restricted their focus to radiographic fusion rates and general postoperative outcomes. Such endpoints, though clinically relevant, provide an incomplete picture of patient recovery and functional improvement. Additionally, these studies were conducted several years ago and relied primarily on absolute outcomes without calculating relative mean differences. This limits their ability to calculate the magnitude of risk in a way that is directly comparable across populations and clinically interpretable. Incorporating relative values, as we have done provides a clearer understanding of smoking’s impact on spinal fusion outcomes. Our study also aimed to expand the scope by incorporating patient-reported outcomes while also including the most recently published literature and assessing fusion and clinical outcomes. Furthermore, we aim to provide more granular data by stratifying patients based on the levels operated on and by distinguishing between former and never smokers.

## Materials and methods

This systematic review and meta-analysis were conducted in accordance with the 2020 Preferred Reporting Items for Systematic Reviews and Meta-Analyses (PRISMA) [22]. The protocol was not registered in a public database.

### Search strategy and criteria

PubMed, EMBASE and Cochrane were searched up to August 2024. Search terms related to cigarette smoking and spinal fusion were used to identify studies analyzing the effect of smoking versus non-smoking and/or smoking cessation on the clinical and radiological outcome of spine surgery (S1 Appendix). Covidence [23] was utilized for the management of articles and literature for the current study.

Studies were included if they met the following criteria: (1) the patient population consisted of patients who underwent spinal instrumented spondylodesis procedure, (2) smoking was the main exposure in the study and/or patients were required to stop smoking before surgery, (3) duration of follow-up was at least two months.

Articles were excluded if they were not written in English or Dutch, if they were nonhuman studies, non-peer-reviewed, case reports, meta-analyses or systematic reviews. Titles and abstracts were screened independently by two of six authors and a third independent author was consulted in the case of conflicting evaluations. Full texts were independently reviewed by two of six authors. A third author was consulted in the case of conflicting evaluations.

### Extraction of data

For each included study, the following information was extracted: study characteristics (study design, year of publication, country where study was conducted, sample size, and study duration); baseline demographics (mean age, smoking history, gender); surgical information (vertebral column level, surgical approach, and numbers of operated levels); clinical outcomes; radiological postoperative outcomes; and post-operative complications. Clinical outcome data that were extracted included: Oswestry disability index (ODI; ranging from 0–100, a higher score indicating worse functionality), Neck Disability Index (NDI; ranging from 0–100, a higher score indicating worse functionality), Visual Analog Scale (VAS) for neck pain, arm pain, back pain, and leg pain (ranging from 0–10; a higher score indicating more pain), EuroQol five-dimension scale (EQ5D; ranging from 0 to 1; a higher score indicating more quality of life), Medical Outcomes Study Questionnaire (SF12; ranging from 0–100, a higher score indicating better health), Japanese Orthopaedics Association score (JOA; ranging from 0 to 17; a higher score indicating better function) and patient satisfaction score measured by the 7 point Likert perceived recovery scale (higher score indicating less satisfaction with outcome). Furthermore, surgical site infections (SSI) and number of reoperations were extracted. The radiological parameters that were extracted concerned bony fusion and pseudoarthrosis rates. Data collection was performed by two of four authors and a third author solved any discrepancies

### Quality assessment

Two authors evaluated the quality of each included study using the Newcastle Ottawa Scale for assessing cohort studies and the Joanna Briggs Institute Appraisal checklist for case series [24,25]. The Newcastle Ottawa Scale assesses three domains: subject selection, comparability, and assessment of outcome, with a total of nine possible points. The scores were categorized as follows: 0–3 points as poor quality; 4–6 points as fair quality; and 7–9 points as good quality. For case series, 10 questions from the checklist were addressed of which there were four possible answers, that is, yes (Y), no (N), unclear (U) or not applicable (NA). Values for each answer included: Y = 1, N, U, NA = 0. The quality scores were thus calculated as follows: 1–4 as low, 5–7 as moderate, and 8–10 as good. In case of any disagreements, the authors discussed and resolved them among themselves. If a consensus could not be reached, a third author provided the final judgment.

### Statistical analysis

The studies reported raw means along with the difference in means for various continuous outcomes. To make for a fair comparison between smokers and non-smokers and to take into account varying baseline values, we calculated the percent mean change from baseline {[(pre-post)/pre]*100%} for each study. For categorical outcomes, the incidence of events along with the 95% confidence interval was derived from each study. For each of the smokers and non-smokers subgroups, patients were further stratified by region of operation (cervical, thoracic, lumbar) or the number of levels operated on (single, multiple) only for those studies that presented these stratified data. Studies that included patients with single or multiple levels of operation as one category could not be represented in this sub-stratified analysis by level of operation. For the main analysis by smokers vs. non-smokers, because former smokers were included among the non-smokers, we further stratified non-smokers into former smokers and never smokers when data were provided by the original studies. All of the pooled point estimates for the reported clinical outcomes and their respective 95% confidence intervals were calculated using a random effects model via the Dersimonian and Laird method [26]. Notably, a direct comparison between outcomes in smokers vs. non-smokers was not possible with the data provided by the individual articles, as no adjustment for confounding was made to compare these two groups. Providing a p-value for such a comparison would be misleading, as these p-values would be biased. Hence, pooled point estimates were provided for each stratum along with their respective 95% CIs as a measure of variability around the point estimates. Forest plots were used to visualize individual and summary estimates. The Higgins I^2^ index was used to evaluate heterogeneity [27]. An I^2^ value of more than 50% was considered high. Due to the small number of studies (< 10) included per analysis, it was not possible to assess the potential for small study effects through funnel plots. Comprehensive Meta-analysis, version 4 (Biostat, Inc, Englewood, New Jersey) was utilized to perform statistical analysis.

## Results

**Fig 1:**
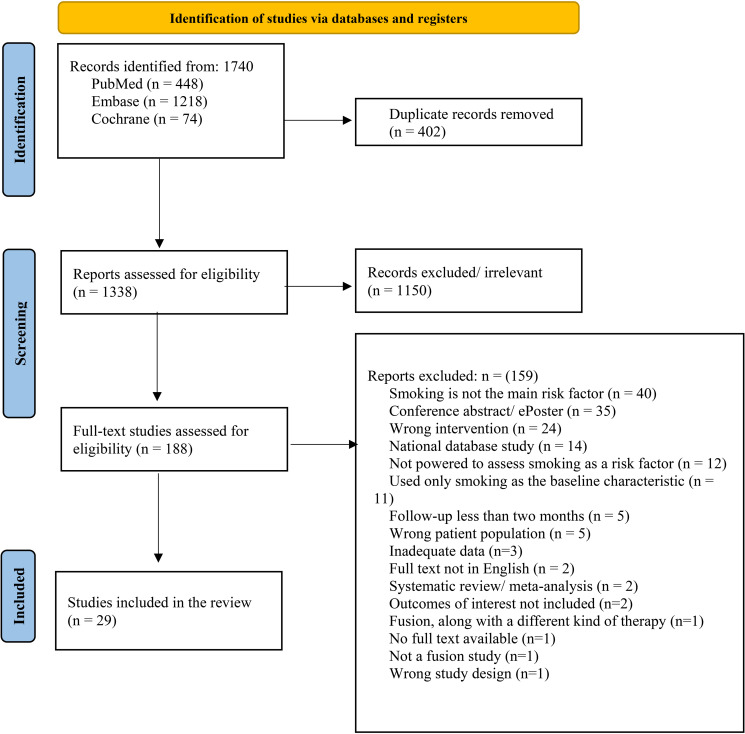
PRISMA flowchart of study selection. This figure provides an overview of the inclusion and exclusion of studies in this review of literature.

There were 1093 articles identified from PubMed, COCHRANE, and Embase databases. After title and abstract screening, 176 articles were found to be eligible for full-text screening and subsequent assessment yielded 29 relevant articles that were included in this study (Fig 1) [10,13,28–54]. This included 27 cohort studies [10,13,29–53] and 2 case series [28,54]. The total sample size of these studies was 6687 patients undergoing various spinal procedures, including both multi-level and single-level procedures in the cervical and lumbar spine. Details of the studies included are listed in Table 1.

**Table 1:** Baseline population characteristics of studies included in the meta-analysis that compared/ included smokers and non-smokers.

First author, publication year	Sample Size		Male: Female	Age (mean)	Follow-up (months)	Surgery region	Surgical approach	Clinical outcomes	Complications	Radiological outcomes	Study quality assessment score†
	Smokers (n)	Non-smokers (n)									
Retrospective cohorts											
*Brown et al., 1986	46	60	50:50	59.0	12	Lumbar	Posterior	N/A	N/A	Pseudoarthrosis	Fair 6
*Glassman et al., 2000	188	169	202:155	44.2	24	Lumbar	Posterior	Patient satisfaction	N/A	Pseudoarthrosis	Good 7
Hilibrand et al., 2001	34	110	N/A	N/A	68	Cervical	Anterior	N/A	N/A	Fusion	Fair 6
Eubanks et al., 2011	41	117	93:55	59.2	15	Cervical	Posterior	N/A	SSI	Fusion	Fair 5
Luca et al, 2011	29	78	76:44	62.2	24	Lumbar	Posterior	N/A	N/A	N/A	Good 7
*Luszczyk et al., 2013	156	417	N/A	N/A	24	Cervical	Anterior	N/A	N/A	Fusion, pseudoarthrosis	Fair 5
*Bydon et al., 2014	50	231	125:156	49.9	53.5	Lumbar	Posterior	N/A	SSI	Pseudoarthrosis	Good 7
*Lau et al., 2014	40	120	93:67	53.1	12	Cervical	Anterior	N/A	SSI	Pseudoarthrosis	Good 8
Kusin et al., 2015	47	87	N/A	N/A	24	Cervical	Anterior and posterior	N/A	N/A	N/A	Good 9
*Hermann et al., 2016	16	34	23:27	53.0	12	Lumbar	Posterior	ODI, VAS back	N/A	Fusion, pseudoarthrosis	Fair 5
Macki et al., 2017	28	82	47:63	N/A	59	Lumbar	Posterior	N/A	SSI	N/A	Good 7
Joswig et al., 2017	96	279	N/A	N/A	12	Lumbar	Posterior	N/A	N/A	N/A	Good 8
*Phan et al., 2017	23	114	65:72	55.2	12	Lumbar	Anterior	ODI, SF-12, patient satisfaction	SSI	Pseudoarthrosis	Fair 6
*Jazini et al., 2018	76	495	N/A	N/A	12	Lumbar	N/A	ODI, VAS back, VAS leg	N/A	N/A	Fair 6
Cerier et al., 2019	23	38	32:29	58.2	12	Cervical	Anterior	NDI	N/A	Fusion	Fair 6
Patel et al., 2019	25	167	115:77	48.7	6	Cervical	Anterior	NDI, VAS neck, VAS arm	N/A	N/A	Fair 6
Tu et al., 2019	20	89	56:53	45	24	Cervical	Anterior	NDI, JOA score, VAS neck, VAS arm	SSI	N/A	Fair 6
Kuo et al., 2020	34	272	166:63	60.2	44	Lumbar	Posterior	JOA score, VAS back	N/A	N/A	Fair 6
Nagoshi et al., 2020	182	405	391:196	66.8	12	Cervical	Posterior	JOA score, VAS neck	SSI	N/A	Good 9
Wen-Shen et al., 2020	20	117	66:71	44.5	24	Cervical	Anterior	N/A	N/A	N/A	Good 7
*Goyal et al., 2021	52	314	202:134	62.4	14	Lumbar	Anterior and Posterior	ODI, SF-12, VAS back, VAS leg	N/A	N/A	Good 8
*Mangan et al., 2021	43	221	123:141	53.0	20	Cervical	Anterior	NDI, VAS neck, VAS arm,	N/A	Pseudoarthrosis	Good 9
Senker et al., 2021	49	138	72:115	64.3	N/A	Minimally invasive spinal fusion surgery		N/A	N/A	N/A	Poor 2
*Wang et al., 2021	40	113	51:102	50.11	24	Cervical	Anterior	NDI, JOA score, VAS neck, VAS arm	N/A	Fusion	Good 9
*Gatot et al., 2022	25	162	62:125	57.6	24	Lumbar	Posterior	ODI, VAS back, VAS leg	N/A	Fusion, pseudoarthrosis	Good 9
*Toci et al., 2022	43	152	120:75	61.5	12	Cervical	Posterior	NDI, JOA score, VAS neck, VAS arm	N/A	N/A	Good 9
±Kruk et al., 2024	16	81	39:58	55	21	Cervical	Anterior	N/A	N/A	Fusion	Good 7
Case series											
±Bose et al., 2001	46	60	47:59	50.1	12	Cervical	Anterior	N/A	N/A	Fusion	Moderate 7
±Bertagnoli et al., 2006	34	70	N/A	47.5	24	Lumbar	Anterior	ODI, VAS back	N/A	N/A	Moderate 5

Abbreviations: N/A = not available, NDI = neck disability index, ODI = Oswestry Disability Index, SF-12 = Short form 12, JOA score = Japanese orthopedic association score, VAS = visual analog scale.

*Indicate studies that stratified non-smokers into former smokers and never smokers.

†Cohort studies were rated based on the Newcastle Ottawa Scale, while case series were rated based on the Joanna Briggs Institute Critical Appraisal Checklist. Of note, none of the retrospective cohort studies adjusted for confounding variables.

±Bose B, 2001, and Kruk MD, 2024 were retrospective case series, while Bertagnoli R, 2006, was a prospective case series.

Of the 29 included studies on the association between smoking and treatment outcomes, seven studies reported the difference in complications following spinal instrumented procedures [13,31,40,43,45,47,50], 15 studies reported radiological outcomes [10,13,28–32,34,35,40,42,44,47,51,53], and 14 reported clinical outcomes [10,30,32–34,36,44–47,49–51,54].

Of the cohort studies, 15 were of good quality [10,13,32,33,37,39–41,43–45,49,51–53], 11 were of fair quality [29–31,34–36,38,42,46,47,50], and one was of poor quality [48] (S2, S3 Appendix). Two of the case series earned a moderate score [28,54] (S4, S5 Appendix). Factors that played a role in lower study quality included studies that did not adjust for potential confounders (including age, sex, number of levels fused, surgery type) and an absence of stratification (of non-smokers into former smokers and never smokers) across each included study (Table 1).

### NDI

Six studies evaluated the differences in NDI (cervical spine) pre- and post-operatively in smokers and non-smokers (S1 Table), and in two studies patients were stratified as smokers, former smokers, and never smokers [44,49]. The pooled percent mean change from baseline for smokers was 42.8% (95% CI: 25.4% to 60.1%; I^2^: 100%; six studies) and for non-smokers was 49.7% (95% CI: 32.4% to 64.0%; I^2^: 100%; six studies) (Table 2). Further subgrouping provided by two out of the six studies resulted in pooled percent mean changes from baseline of 32.6% (95% CI: 11.1% to 54.1%; I^2^: 100%) in never-smokers, 22.7% (95% CI: 1.2% to 44.2%; I^2^: 100%) in former smokers, and 24.2% (95% CI: 2.7% to 45.7%; I^2^: 100%) in smokers (S2 Table). The results of the subgroup analysis suggested a numerically better outcome in never-smokers, though this may not be clinically important (Table 3). Only two studies distinguished between the number of levels fused (multilevel surgery) and stratification revealed a consistent improvement in NDI amongst non-smokers (Table 2).

**Table 2:** Pooled point estimates of various outcomes (surgical site infection, reoperation, fusion, pseudoarthrosis, NDI, ODI, SF-12, Joa score, VAS back, leg, neck, and arm pain, patient satisfaction) for smokers and non-smokers^±^.

Outcome (number of studies – n)	Number of patients, overall, and stratified by smoking status	Smokers Pooled point estimates (95% Confidence interval)	Non-smokers Pooled point estimates (95% Confidence interval)
**Pooled percent mean change from baseline†**			
NDI* Overall (n = 6) Multi-level (n = 2)	**Overall: 974** Smokers: 194 Non-smokers: 780	**42.8% (25.4 to 60.1)** 49.9% (6.4 to 93.4)	**49.7% (32.4 to 64.0)** 70.0% (61.0 to 79.0)
*ODIǂ* **Overall (6)** Multi-level (n = 1) Single level (n = 1)	**Overall: 1415** Smokers: 226 Non-smokers: 1189	**48.6% (34.7 to 62.5)** 60.0% (59.9 to 60.1) 72.1% (71.9 to 72.3)	**56.5% (42.6 to 70.3)** 66.2% (66.1 to 66.3) 77.8% (77.8 to 77.8)
VAS neck pain* Overall (n = 6) Multi-level (n = 1)	**Overall: 1090** Smokers: 254 Non-smokers: 836	**53.3% (39.6 to 66.9)** 75.0% (75.0 to 75.0)	**49.6% (35.9 to 63.2)** 74.1% (74.1 to 74.1)
VAS arm pain* Overall (n = 5) Multi-level (n = 1)	**Overall: 913** Smokers: 171 Non-smokers: 742	**53.6% (43.6 to 63.5)** 44.8% (44.8 to 44.8)	**54.7% (44.8 to 64.4)** 71.4% (71.4 to 71.4)
*VAS back painǂ* **Overall (n = 5)** Multi-level (n = 1) Single level (n = 1)	**Overall: 1278** Smokers: 203 Non-smokers: 1075	**55.0% (29.7 to 80.2)** 61.0% (61.0 to 61.0) 78.3% (78.1 to 78.5)	**60.5% (35.2 to 85.7)** 55.6% (55.6 to 55.6) 88.5% (88.5 to 88.5)
VAS leg pain*ǂ* **Overall (n = 3)** Single level (n = 1)	**Overall: 1124** Smokers: 153 Non-smokers: 971	**70.8% (42.3 to 99.3)** 84.3% (84.1 to 84.5)	**70.6% (42.1 to 99.1)** 93.1% (93.1 to 93.1)
JOA score* Overall (n = 4) Multi-level (n = 1)	**Overall: 757** Smokers: 250 Non-smokers: 507	**25.3% (8.4 to 42.2)** 47.2% (47.1 to 47.3)	**21.4% (4.5 to 38.3)** 45.0% (45.0 to 45.0)
**Pooled mean score post-surgery**			
*SF-12* **Overall (n = 2)**	**Overall: 503** Smokers: 75 Non-smokers: 428	**38.07 (35.84 to 40.30)**	**40.31 (39.14 to 41.47)**
*Patient satisfaction* **Overall (n = 2)**	**Overall: 494** Smokers: 211 Non-smokers: 283	**2.35 (1.51 to 3.19)**	**2.05 (1.21 to 2.88)**
**Pooled incidence††**			
*Fusion* **Overall (n = 9)** Multi-level (n = 6) Single level (n = 2) Cervical Spine (n = 7) Lumbar Spine (n = 2)	**Overall: 1445/1575** Smokers: 363/418 Non-smokers: 1082/1157	**88.2% (78.0 to 94.0)** 87.5% (72.0 to 95.0) 91.2% (86.0 to 94.5) 87.0% (73.4 to 94.2) 86.8% (71.9 to 94.4)	**94.9% (89.9 to 97.5)** 94.7% (86.8 to 97.9) 96.3% (79.1 to 99.5) 94.2% (89.0 to 97.0) 97.3% (88.2 to 99.4)
*Pseudoarthrosis* **Overall (n = 9)** Multi-level (n = 2) Single level (n = 2) Cervical Spine (n = 3) Lumbar Spine (n = 6)	**Overall: 216/2081** Smokers: 97/576 Non-smokers: 119/1505	**17.2% (11.7 to 24.4)** 31.4% (14.5 to 55.3) 8.8% (5.5 to 14.0) 9.2% (4.4 to 18.0) 6.3% (3.3 to 11.7)	**7.3% (4.9 to 10.7)** 7.2% (3.3% to 15.1) 3.7% (0.5 to 20.9) 8.3% (6.5 to 10.5) 8.8% (5.5 to 14.0)
*Surgical site infection* **Overall (n = 7)** Multi-level (n = 1) Cervical Spine (n = 4) Lumbar Spine (n = 3)	**Overall: 31/1271** Smokers: 11/360 Non-smokers: 20/911	**4.6% (2.5 to 8.5)** 2.4% (0.3 to 15.4) 3.5% (1.0 to 11.7) 4.8% (1.9 to 11.5)	**2.6% (1.6 to 4.3)** 0.0% (0.0 to 0.1) 1.4% (0.6 to 3.1) 3.6% (2.2 to 5.9)
*Reoperations* **Overall (n = 4)** Multi-level (n = 1) Cervical Spine (n = 3) Lumbar Spine (n = 1)	**Overall: 48/589** Smokers: 14/137 Non-smokers: 34/452	**10.4% (4.1 to 24.1)** 4.3% (1.1 to 15.8) 5.3% (2.3 to 11.7) 32.1% (17.6 to 51.1)	**7.5% (3.2 to 16.7)** 6.7% (2.5 to 16.5) 6.3% (2.8 to 13.7) 13.4% (7.6 to 22.6)
*Patient satisfaction dichotomous* **Overall (n = 2)**	**Overall: 264/291** Smokers: 51/59 Non-smokers: 213/232	**86% (65–95)**	**91% (79–97)**

Abbreviations: NDI = neck disability index; ODI = Oswestry Disability Index; SF-12 = Short form 12; JOA score = Japanese orthopedic association score; VAS = visual analog scale.

†† Incidence was derived from a ratio consisting of a numerator (number of cases) and a denominator (sample size).

† Percent mean change from baseline is calculated as {[(pre-post)/ pre)]*100%}; a greater percentage indicates a greater change and thus a better outcome.

* Study only included patients undergoing cervical spine surgery.

ǂ Study only included patients undergoing lumbar spine surgery.

**Bold** font indicates overall pooled point estimates without stratification.

**Table 3:** Pooled point estimates of various outcomes (pseudoarthrosis, NDI, ODI, Joa score, VAS back, leg, neck, and arm pain) stratified by smoking history (smokers, former smokers, and never smokers) undergoing spondylodesis.

Outcome (n = number of patients)	Pooled point estimates (95% Confidence interval)	Number of studies (n)	I^2^(%)
*NDI (n = 459)* Smokers (n = 86) former smokers (n = 120) never smokers (n = 253)	*Pooled PMC from baseline†* 24.2% (2.7 to 45.7) 22.7% (1.2 to 44.2) 32.6% (11.1 to 54.1)	2	100 100 100
*ODI (n = 937)* Smokers (n = 128) former smokers (n = 282) never smokers (n = 527)	*Pooled PMC from baseline†* 30.4% (17.6 to 43.2) 39.9% (27.0 to 52.7) 47.7% (34.8 to 60.5)	2	100 100 100
*VAS neck pain (n = 612)* smokers (n = 126) former smokers (n = 154) never smokers (n = 332)	*Pooled PMC from baseline†* 56.8% (35.2 to 78.5) 54.5% (32.9 to 76.1) 53.8% (32.1 to 75.4)	3	100 100 100
*VAS arm pain (n = 612)* smokers (n = 126) former smokers (n = 154) never smokers (n = 332)	*Pooled PMC from baseline†* 59.8% (41.8 to 77.8) 61.9% (43.8 to 79.9) 54.8% (36.8 to 72.9)	3	99.9 100 100
*VAS Back pain (n = 937)* smokers (n = 128) former smokers (n = 282) never smokers (n = 527)	*Pooled PMC from baseline†* 40.3% (29.8 to 50.7) 42.8% (32.3 to 53.2) 51.6% (41.1 to 62.1)	2	100 100 100
*VAS Leg pain (n = 937)* smokers (n = 128) former smokers (n = 282) never smokers (n = 527)	*Pooled PMC from baseline†* 38.1% (26.0 to 50.2) 54.6% (42.4 to 66.7) 62.2% (50.0 to 74.3)	2	100 100 100
*JOA score (n = 348)* Smokers (n = 83) former smokers (n = 85) non-smokers (n = 180)	*Pooled PMC from baseline†* 26.9% (−19.9 to 73.7) 22.6% (−24.2 to 69.3) 27.2% (−19.6 to 74.0)	2	100 100 100
*Pseudoarthrosis (n = 31/396)* Smokers (5/68) former smokers (17/222) never smokers (9/106)	*Pooled incidence*†† 9.5% (3.2 to 25.0) 8.5% (3.5 to 18.8) 7.2% (3.3 to 15.0)	2	69.4 0.00 36.2

Abbreviations: PMC = Percent mean change; NDI = neck disability index; ODI = Oswestry Disability Index; SF-12 = Short form 12

JOA score = Japanese orthopedic association score; VAS = visual analog scale. † Relative mean difference from baseline is calculated as (pre-post/pre)

†† Incidence was derived from a ratio consisting of a numerator (number of cases) and a denominator (sample size).

### ODI

The change in ODI (lumbar spine) following surgery was determined in smokers and non-smokers in six different studies. This was followed by a subgroup analysis consisting of smokers, former smokers and never smokers in two studies [33,36]. The pooled percent mean change from baseline was 48.6% (95% CI: 34.7% to 62.5%; I^2^: 100%) in smokers and 56.5% (95% CI: 42.6% to 70.3%; I^2^: 100%) in non-smokers (Table 2, S3 Table), indicating a greater numerical decrease in ODI in non-smokers. Furthermore, the sub-analysis performed in two [33,36] out of six studies showed incrementally increased percent mean changes from smokers: 30.4% (95% CI: 17.6% to 43.2%; I^2^: 100%), to former smokers: 39.9% (95% CI: 27.0% to 52.7; I^2^: 100%), and finally never smokers: 47.7% (95% CI: 34.8% to 60.5%; I^2^: 100%) (Table 3, S4 Table). Two studies distinguished the number of levels fused with only one study exclusively studying single-level fusion and the other multi-level fusion; stratification revealed similar results, that is, apparent better ODI scores amongst non-smokers (Table 2).

### VAS neck pain

Neck pain was recorded using VAS (cervical spine) in six articles [44–46,49–51]. The pooled percent mean change from baseline was 53.3% (95% CI: 39.6% to 66.9%; I^2^: 100%) for smokers and 49.6% (95% CI: 35.9% to 63.2%; I^2^: 100%) for non-smokers (Table 2; S5 Table). Non-smokers were divided into former-smokers and never-smokers in three [44,49,51] out of the six studies, yielding 56.8% (95% CI: 35.2%to 78.5%; I^2^: 100%) for smokers, 54.5% (95% CI: 32.9% to 76.1%; I^2^: 100%) for former-smokers, and 53.8% (95% CI: 32.1% to 75.4%; I^2^: 100%) for never-smokers (Table 3, S6 Table). One study limited their data to patients with fusion across multiple levels and upon stratification, demonstrating that, similar to the pooled results, there was a comparable change in the VAS neck pain scores (Table 2).

### VAS arm pain

VAS arm pain was assessed in five studies focusing on cervical spine [44,46,49–51]. The pooled percent mean changes were 53.6% (95% CI: 43.6% to 63.5%; I^2^: 100%) for smokers and 54.7% (95% CI: 44.8% to 64.4%; I^2^: 100%) for non-smokers (Table 2; S7 Table). On further stratification of non-smokers into former-smokers and never-smokers for three [44,49,51] out of the five studies, a comparable result between groups was obtained: a decrease of 59.8% (95% CI: 41.8% to 77.8%; I^2^: 99.9%) for smokers, 61.9% (95% CI: 43.8% to 79.9%; I^2^: 100%) for former-smokers, and 54.8% (95% CI: 36.8% to 72.9%; I^2^: 100%) for never-smokers (Table 3, S8 Table). Only one study exclusively evaluated multi-level fusions and found that non-smokers showed a larger decrease in arm pain (Table 2).

### VAS back pain

Clinical outcomes on back pain scores were assessed in five articles [32–34,36,54] that studied lumbar spine surgeries. Pooled percent mean change from baseline was 55.0% (95% CI: 29.7% to 80.2%; I^2^: 100%) for smokers, and 60.5% (95% CI: 35.2% to 85.7%; I^2^: 100%) for non-smokers (Table 2; S9 Table). Further subgrouping for two [33,36] out of the five studies resulted in pooled percent mean changes of 40.3% (95% CI: 29.8% to 50.7%; I^2^: 100%) for smokers, 42.8% (95% CI: 32.3% to 53.2%; I^2^: 100%) for former-smokers, and 51.6% (95% CI: 41.1% to 62.1%; I^2^: 100%) for never-smokers (Table 3; S10 Table). One study evaluated multi-level fusions only, demonstrating similar results. Another study evaluated single-level fusions only and revealed that non-smokers showed more improvement than smokers, without overlapping confidence intervals. (Table 2).

### VAS leg pain

VAS leg pain was assessed in three articles (lumbar spine) [32,33,36]. Pooled percent mean change from baseline was 70.8% (95% CI: 42.3% to 99.3%; I^2^: 100%) for smokers and a comparable 70.6% (95% CI: 42.1% to 99.1%; I^2^: 100%) for non-smokers (Table 2; S11 Table). Two [33,36] out of the three studies provided data on three categories of smoking history. Further stratification revealed worse values for smokers 38.1% (95% CI: 26.0% to 50.2%; I^2^: 100%) and former-smokers 54.6% (95% CI: 42.4% to 66.7%; I^2^: 100%) compared to never-smokers 62.2% (95% CI: 50.0% to 74.3%; I^2^: 100%) (Table 3; S12 Table). It should be noted that one of the studies did not have enough data available to be utilized in the sub-analysis; this study demonstrated a larger decrease in the outcome (over 80%) compared to the other two studies and had a smaller sample size. This discrepancy should be considered when interpreting the subgroup results. One study also evaluated single-level fusion only and revealed that non-smokers displayed a greater improvement in VAS leg pain, without overlapping confidence intervals. (Table 2).

### JOA score

The percent mean change from baseline for JOA scores was derived from four studies (cervical spine) [45,49–51]. The pooled percent mean change was 25.3% (95% CI: 8.4% to 42.2%; I^2^: 100%) for smokers and 21.4% (95% CI: 4.5% to 38.3%; I^2^: 100%) for non-smokers Table 2; S13 Table. Further stratification by two [49,51] out of the four studies revealed values of 26.9% (95% CI: −19.9% to 73.7%; I^2^: 100%) for smokers, 22.6% (95% CI: −24.2% to 69.3%; I^2^: 100%) for former-smokers, and 27.2% (95% CI: −19.6% to 74.0%; I^2^: 100%) for never-smokers (Table 3; S14 Table). Stratification based on the number of levels operated on revealed one study that assessed multi-level fusions, demonstrating a trend similar to the overall pooled mean difference (Table 2).

### Fusion

Nine articles [28,30–32,34,35,42,51,53] described fusion rates; the pooled incidence was 86.8% (95% CI: 75.9%−93.2%; I^2^: 77.8%) in smokers vs. 95.1% (95% CI: 91.0%−97.4%; I^2^: 78.5%) in non-smokers (S6 Appendix). Stratification based on the region, that is, cervical (seven studies), lumbar (two studies), and number of levels operated on, that is, single level (two studies) and multi-level (six studies), displayed the same trend of a higher number of patients demonstrating fusion in non-smokers (Table 2).

### Pseudoarthrosis

Likewise, nine articles [10,13,29,32,34,40,42,44,47] described the occurrence of pseudoarthrosis. The pooled incidence of pseudoarthrosis was 17.2% (95% CI: 11.7% to 24.4%; I^2^: 73.6%) in smokers, 7.3% (95% CI: 4.9% to 10.7%; I^2^: 64.2%) in non-smokers (S7 Appendix). Subgroup analyses by two [40,44] out of the nine studies yielded a pooled incidence of pseudoarthrosis in 9.5% of participants who were smokers (95% CI: 3.2% to 25.0%; I^2^: 73.6%), 8.5% of former-smokers (95% CI: 3.5% to 18.8%; I^2^: 0.00%), and 7.2% of never-smokers (95% CI: 3.3% to 15.0%; I^2^: 36.2%) (S8 Appendix). Studies focusing exclusively on multi-level fusion demonstrated more pseudoarthrosis in smokers, but a difference was absent in studies focusing on mono-level fusion. Upon stratification based on regions, comparable results were shown for smokers and non-smokers (Table 2).

### Patient satisfaction

Patient satisfaction was analyzed in two studies [10,47] as a mean score (continuous outcome) between smokers and non-smokers. The pooled post-surgical mean score for patients’ satisfaction was 2.35 (95% CI: 1.51 to 3.19; I^2^: 96.3%) for smokers and 2.05 (95% CI: 1.21 to 2.88; I^2^: 97.6%) for non-smokers (S9 Appendix). Another two studies [32,54] reported patient satisfaction as a binary outcome (1 and 2: good result, 3–7: bad result) for smokers and non-smokers. Surprisingly, the pooled incidence for the good results was 86% (95% CI: 65% to 91%; I^2^: 71.4%) in smokers and 91% (95% CI: 79% to 97%; I^2^: 64.1%) in non-smokers (S10 Appendix).

### Surgical site infection

Seven articles [13,31,40,43,45,47,50] accounted for surgical site infections (SSI). The pooled incidence of SSI was 4.6% in smokers (95% CI: 2.5%−8.5%; I^2^: 22.5%) vs. 2.6% (95% CI: 1.6%−4.3%; I^2^: 6.00%) in non-smokers (S11 Appendix). The pooled incidences of SSI in non-smokers remained relatively lower than in smokers for each of the multi-level groups, cervical spine group, and lumbar spine group (Table 2).

### Reoperations

Four studies [28,43,44,50] provided data on reoperations. The pooled incidence of reoperations was 10.4% (95% CI: 4.1%−24.1%; I^2^: 77.7%) in smokers vs. 7.5% (95% CI: 3.2%−16.7%; I^2^: 53.46%) in non-smokers (S12 Appendix). One study evaluating reoperations after lumbar spine fusion surgery demonstrated convincingly more reoperations in smokers (Table 2).

### SF-12

Amongst two studies [33,47], the change in SF-12 between smokers and non-smokers were comparable. The mean post-operative SF-12 score was 38.07 (95% CI: 35.84 to 40.30; I^2^: 0.00%) for smokers and 40.31 (95% CI: 39.14 to 41.47; I^2^: 44.6%) for non-smokers (Table 2; (S13 Appendix). A sub-analysis could not be done for this outcome measure due to a lack of stratified results among studies.

Across outcomes, both smokers and non-smokers demonstrated clinically meaningful improvements in pain and function; however, between smokers and non-smokers, the differences were generally small and often not clinically significant. For VAS back and leg pain, ODI, NDI, and JOA scores, smokers and non-smokers achieved comparable improvements, with subgroup differences showing wide confidence intervals and uncertain clinical importance. In contrast, structural outcomes demonstrated more clinically relevant differences. For SSI and reoperations, the absolute differences were smaller but still potentially meaningful given the morbidity of these events.

## Discussion

Current results demonstrated a trend for inferior outcomes among smokers across some of the recorded clinical outcomes. In some cases, although a numerical difference was seen, this was not always suggestive of a clinically relevant difference. Stratification into the categories of smokers, former smokers and never-smokers aligned with our hypothesis of smoking being inversely associated with clinical outcomes in patients undergoing instrumented spondylodesis surgery in some outcomes.

Notably, of the studies included, none of the so-called ‘cohort’ studies properly adjusted for confounding in these observational studies. Some studies [13,30,31,36] relied solely on conducting an unpaired t-test or an analysis of variance to compare the mean outcomes across the groups; yet, these analyses are known to be inappropriate from an epidemiological standpoint for cohort study designs, a type of analytical observational study. None of these studies adjusted for confounding through matching or statistical analysis. Therefore, in this analysis, we had to treat each of these so-called “cohort” studies as two separate case series, one on smokers and another on non-smokers.

Critical and fundamental confounders such as age, sex, number of levels fused, and surgical approach are well-established determinants of both fusion success and patient-reported outcomes; their omission introduces substantial risk of residual confounding. (Cite) It is thus difficult to independently observe smoking and disregard these clinical and demographic factors. The apparent results reported in prior studies may be an underestimation or an overestimation of the effect of smoking. This greatly limits the strength of the findings from this study; however, it provides incentive for future studies to take into consideration confounders to provide a more accurate perspective of the deleterious effects of smoking on spondylodesis.

Some clinical outcomes displayed a hierarchical pattern, whereby never-smokers demonstrated the most favorable results, followed by former-smokers, and subsequently smokers. This suggests that relying solely on current smoking status may not provide a comprehensive insight into patient outcomes, as demonstrated by poorer clinical outcomes in former smokers relative to their never-smoker counterparts. Our findings highlight the importance of early smoking cessation ahead of surgical intervention; proactive measures have the potential to ameliorate the incidence of negative outcomes, disability, and pain. By recognizing the continuum of smoking impact, encompassing current smokers, former smokers, and even those with a remote history of smoking, healthcare providers are prompted to engage in more targeted interventions to enhance patient outcomes following spinal fusion procedures, including encouraging current smokers to stop smoking as early as possible before surgical intervention.

Our study took the step to further analyze patients based on the region of surgery (that is, cervical, thoracic, and lumbar spine) and the number of levels operated on (that is, multilevel or single-level fusion). However, only a limited number of studies made a meaningful distinction. Even so, we observed the same trend upon sub-analysis allowing us to formulate our hypothesis that patients who undergo spondylodesis, irrespective of the number of levels operated on or the region of operation, have poorer outcomes after surgery, with a history of smoking, may it be active or a remote. Better adjusted comparative studies could provide us with data to further test and substantiate this hypothesis.

A meta-analysis conducted in 2016, focusing on the impact of smoking in the context of spinal procedures, explored the risk posed by smoking in relation to surgical site infections (SSIs). In congruence with our present investigation, the work by Kong et al. similarly demonstrated a direct association between smoking and the incidence of SSIs, with smokers displaying a higher incidence of SSI when compared to non-smokers [19]. Kong and colleagues drew attention to the possibility of false negatives in their analysis, attributing these findings to the inherent heterogeneity present across the included studies [19]. Consistent with the observations of Kong et al., our current study also encountered substantial heterogeneity among the included studies, with most outcomes having an I^2^ value of 100%. The substantial heterogeneity limits the interpretability of our findings and thus, results should be viewed with caution. The observed heterogeneity could be attributed to the wide variability in baseline characteristics and the diverse spectrum of procedure levels encompassed by the studies, spanning multi-level and single-level procedures as well as cervical and thoracic. This intrinsic variation in study characteristics may have contributed to the marked clinical and statistical heterogeneity observed across the pooled data. Acknowledging this heterogeneity is crucial for an accurate interpretation of the findings, highlighting the need for caution when drawing conclusions from the results. In contrast to the study conducted by Kong, however, we adopted a holistic approach to analyzing post-operative outcomes, expanding our study to include clinical outcomes, radiological outcomes, and complications. Additionally, we also stratified non-smokers into former smokers and never smokers, providing greater depth and understanding to our analysis [19].

In a more recent meta-analysis conducted in 2021, the primary focus was directed towards comprehending the ramifications of smoking on fusion rates after spinal interventions. Distinguishing itself from preceding investigations, this study notably pursued a stratified approach, dividing outcomes into the categories of single-level and multi-level fusions. Similar to our own inquiry, the findings of this systematic review noted a discernible inverse relationship between smoking and fusion rates, indicating a reduced likelihood of fusion success among smokers [17]. Our results were also in line with previous reports on the effects of smoking on non-union following spinal fusion [16,18] and post-operative adverse events within 90 days [55].

While a systematic review similar to the present study was conducted in 2016 by Jackson et al., many newer studies have emerged since then that have provided more insight into the relationship between smoking and poorer clinical outcomes [30,32,33,36–38,43–52]. Jackson emphasized the subjective and objective outcomes of spine surgery among smokers and among those who stopped smoking prior to surgery [20]. Our study notably took the analytical approach one step further by introducing a finer stratification within the non-smoker cohort. This stratification implies that even a historical context of smoking exerts an influence on adverse outcomes among patients. However, it must be highlighted that the nuanced impact on postoperative outcomes, particularly in the context of patients required to cease smoking before surgery as opposed to those who remain active smokers, remains inadequately characterized within the existing body of literature.

Unlike prior reviews, which have been largely limited to radiographic fusion outcomes, our analysis integrates both structural endpoints and patient-reported outcomes, offering a more comprehensive picture and a patient-centered perspective on the impact of smoking on spinal fusion. We also conducted stratified analyses by smoking history (current, former, and never smokers) as well as by surgical characteristics (single versus multi-level fusion) providing granularity not previously reported. Additionally, we interpreted the findings not only in terms of statistical significance but also in terms of clinical meaningfulness, thereby translating the results into practical terms for patient counseling and surgical planning. This provides important information, that is, although smokers may achieve similar pain and functional recovery in the short-term following surgery (though small differences do exist that are more favorable to non-smokers), this does not translate to improved surgical outcomes in the long-term, with smokers having a higher probability of non-union and requiring reoperation.

Although developing a comprehensive understanding of the temporal dynamics of smoking to optimize outcomes in patients categorized as smokers would be an interesting avenue for future research, the complexities of optimal smoking cessation timelines hinder such studies.

### Limitations and strengths

Notable limitations were variations evident in baseline characteristics among the groups being compared in the studies (smokers, non-smokers), introducing a confounding element that limited our ability to determine the unbiased association between smoking and adverse clinical outcomes. Such variations can be attributed to poor study designs wherein patient cohorts were not matched, nor were there any attempts to adjust for confounding. There was also a significant heterogeneity across most studies, making it difficult to identify trends and associations between different outcome measures and smokers; however, we purposefully did not conduct a statistical analysis that directly compared smokers to non-smokers because of the lack of adjustment for confounding in the original studies. Moreover, to address this heterogeneity, we adhered to robust statistical techniques and performed sub-analyses by distinguishing between the regions where the fusion surgeries were performed and the number of levels that were operated on. In some cases, this reduced the heterogeneity and allowed us to draw more concrete conclusions. Additional data on the timeline of smoking cessation and pack-years of former/current smokers were not available in any of the studies. This may also have impacted the results.

Furthermore, not all of the included studies undertook the distinction between former-smokers and never-smokers within the non-smoker category, which accounted for a lower number of studies for comparison in the sub-analysis. In addition to this, information about the extent of smoking within both the current smoker and former smoker categories in terms of pack-years and time elapsed between smoking cessation and surgery was unavailable. This information could potentially provide more insight into the disparity in outcomes across included studies. Such information may also hold value in guiding personalized mitigation strategies tailored toward patients based on their individual smoking history. Nevertheless, our sub-analysis revealed a hierarchical association between patients with a history of smoking (whether they be former or current smokers) and clinical outcomes.

Despite these limitations, our meta-analysis had several strengths. We calculated the percent mean change from baseline for several outcomes, and we pooled these results, instead of calculating the absolute mean difference, which does not account for the different baseline values across studies. Additionally, in an attempt to reduce heterogeneity and make the most sense of the relation between smoking status and the different clinical outcomes, we stratified our pooled results in several ways: I) By two smoking categories (smokers vs. non-smokers); II) Within each of these categories, we further stratified by different regions of operation and the number of levels operated on; III) We then further stratified the non-smokers into former smokers and never smokers, when such data were provided; steps that were not taken in previously conducted meta-analyses. These statistical methods helped disentangle some of the associations between smoking status and clinical outcomes.

## Conclusions

Our meta-analysis demonstrated that smoking could be associated with poorer clinical and radiological outcomes, in most instances, when compared to non-smokers. Future well-designed comparative studies with appropriate adjustments for confounding are needed to confirm these findings, which have the potential to unveil an unbiased association between smoking (never/former/current) and outcomes following instrumented spondylodesis surgery.

## Supporting information

S1 Appendix Search Terms. (DOCX)

S2 Appendix Coding manual for cohort studies – Newcastle-Ottawa Scale. Description of method used for assessment of risk of bias in cohort studies.
(DOCX)

S3 Appendix Study quality assessment using Newcastle-Ottawa Scale for cohorts – results. Overview of the study quality assessment for included cohort studies.
(DOCX)

S4 Appendix Joanna Briggs Institute Critical Appraisal Checklist for Case Series (2020) – Questions. Description of the method used for assessment of risk of bias in case series.
(DOCX)

S5 Appendix Joanna Briggs Institute Critical Appraisal Checklist for Case Series (2020) – Results. Overview of study quality assessment for included case series.
(DOCX)

S6 Appendix Forest plot depicting event rate of fusion in smokers and non-smokers. (DOCX)

S7 Appendix Forest plot depicting event rate of pseudoarthrosis in smokers and non-smokers. (DOCX)

S8 Appendix Forest plot depicting event rate of pseudoarthrosis in smokers, former smokers and never smokers. (DOCX)

S9 Appendix Forest plot depicting patient satisfaction means in smokers and non-smokers. (DOCX)

S10 Appendix Forest plot depicting patient satisfaction means in dichotomized results. (DOCX)

S11 Appendix Forest plot depicting event rate of surgical site infection in smokers and non-smokers. (DOCX)

S12 Appendix Forest plot depicting event rate of reoperations in smokers and non-smokers. (DOCX)

S13 Appendix Forest plot depicting mean SF-12 score in smokers and non-smokers. (DOCX)

S1 Table Comparison of the difference between mean NDI scores along with the percent mean change for smokers and non-smokers across the original studies. (DOCX)

S2 Table Comparison of the difference between mean NDI scores along with the percent mean change for smokers, former smokers, and never smokers across different studies. (DOCX)

S3 Table Comparison of the difference between mean ODI scores along with the percent mean change for smokers and non-smokers across the original studies. (DOCX)

S4 Table Comparison of the difference between mean ODI scores along with the percent mean change for smokers, former smokers, and never smokers across different studies. (DOCX)

S5 Table Comparison of the difference between VAS neck pain scores along with the percent mean change for smokers and non-smokers across different studies. (DOCX)

S6 Table Comparison of the difference between mean VAS neck pain scores along with the percent mean change for smokers, former smokers, and never smokers across different studies. (DOCX)

S7 Table Comparison of the difference between mean VAS arm pain scores along with the percent mean change for smokers and non-smokers across different studies. (DOCX)

S8 Table Comparison of the difference between mean VAS arm pain scores along with the percent mean change for smokers, former smokers, and never smokers across different studies. (DOCX)

S9 Table Comparison of the difference between mean VAS back pain scores along with the percent mean change for smokers and non-smokers across different studies. (DOCX)

S10 Table Comparison of the difference between mean VAS back pain scores along with the percent mean change for smokers, former smokers, and never smokers across different studies. (DOCX)

S11 Table Comparison of the difference between mean VAS leg pain scores along with the percent mean change across different studies. (DOCX)

S12 Table Comparison of the difference between mean VAS leg pain scores along with the percent mean change for smokers, former smokers, and never smokers across different studies. (DOCX)

S13 Table Comparison of the difference between mean JOA scores along with the percent mean change for smokers and non-smokers across different studies. (DOCX)

S14 Table Comparison of the difference between mean JOA scores along with the percent mean change for smokers, former smokers, and never smokers across different studies. (DOCX)
