# Supplementary material for: The association between smoking and clinical outcomes among spondylodesis patients: A systematic review and meta-analysis
Source: PLoS One. 2026 Jan 13;21(1):e0337799. doi: 10.1371/journal.pone.0337799 (PMC12799005; doi:10.1371/journal.pone.0337799)
Supplement: S1 Appendix — (DOCX) [file pone.0337799.s001.docx]

**Appendix S1: Search Terms**

***PubMed Strategy***

PubMed Search Strategy

**Hits - #448**

Date Run – August 1^st^, 2024

("smoking”[Mesh] OR Tobacco Use [tw] OR Pipe Smoking [tw] OR Smoking Reduction[tw] OR Smoking Cessation [tw] OR Tobacco Use Cessation [tw] OR Tobacco Smok*[tw] OR Cigar Smok*[tw] OR Cigarette Smok*[tw] OR Pipe Smok*[tw] OR Smoking Reduction [tw] OR Smoking Cessation [tw] OR Tobacco Use Cessation [tw] OR Stop* Smoking[tw] OR Give* up Smoking [tw] OR Quit* Smoking [tw] OR Tobacco Consumption [tw] OR Tobacco Chewing [tw] OR Tobacco Smoke Inhalation[tw] OR vaping [tw] OR Vape [tw] OR electronic cigarette* [tw] OR e-cigarette* [tw] OR Smokeless Tobacco Cessation [tw] OR smoking Behaviour* [tw] OR Smoking Habit* [tw] OR Nicotine Use [tw] OR Smoke*[tw] OR Non-smoke* [tw] OR Abstaining smoker* [tw] OR abstinent smoker*[tw] OR ex-smoker* [tw] OR former smoker* [tw] OR past smoker*[tw] OR smoking abstainer* [tw] OR smoking regulation* [tw] OR current smoker*[tw] OR non-smoker* [tw] OR bidi smok* [tw] OR hookah [tw] OR dipping tobacco [tw] OR naswar [tw] OR smoking device* [tw] OR smoking pipe* [tw] OR tobacco snuff [tw] OR vapers [tw] OR smoking abstination [tw] OR Smoking dishabituation [tw] OR nicotine abstination [tw] OR nicotine abstinence [tw] OR nicotine withdrawal [tw] OR Tobacco abstinence [tw] OR nicotine cessation [tw] OR nicotine abstention [tw])

AND

("Spinal Fusion"[Mesh] OR spin* fusion* implant* [tw] OR dorsal spin* fusion* [tw] OR dorsal vertebral fusion* [tw] OR spin* interbody fusion* [tw] OR Spondylodesis [tw] OR vertebral fusion* [tw] OR spin* Arthrodesis [tw] OR spin* fusion* surger* [tw] OR spin* spondylosis surger* [tw] OR anterior spin* fusion* [tw] OR posterior interbody fusion* [tw] OR Posterior spin* fusion* [tw] OR Posterior lumbar interbody fusion* [tw] OR lumbar interbody fusion* [tw] OR ALIF [tw] OR anterior lumbar interbody fusion* [tw] OR PLIF [tw] OR transforaminal interbody fusion* [tw] OR TLIF [tw] OR lateral lumbar interbody fusion* [tw] OR LLIF [tw] OR direct lateral interbody fusion* [tw] OR DLIF [tw] OR Extreme Lateral interbody fusion* [tw] OR XLIF [tw] OR Thoracolumbar fusion* [tw] OR multi-level anterior cervical discectomy [tw] OR lumbar interbody fusion* [tw] OR minimally invasive transforaminal interbody fusion* [tw] OR Spondylodeses [tw] OR Spondylosyndesis [tw] OR spondylosis [tw] OR posterolateral gutter fusion* [tw] OR Multi-level spinal fusion* [tw] OR instrumented spin* fusion* surger* [tw] OR lumbar fusion* [tw] OR anterior interbody fusion* [tw] OR anterior vertebra fusion* [tw] OR anterior vertebral fusion* [tw] OR cervical spin* anterior interbody fusion* [tw] OR spin* anterior fusion* [tw] OR posterolateral spin* fusion* [tw])

#448 hits on January 1^st^, 2023

***EMBASE STRATEGY***

Embase <1974 to 2024 August>

**#1218 Hits**

Embase <1974 to 2024 August>

1 exp "smoking and smoking related phenomena"

2 (Tobacco-use or Pipe Smoking or Smoking Reduction or Smoking Cessation or Tobacco-Use-Cessation or Tobacco Smok* or Cigar Smok* or Cigarette Smok* or Pipe Smok* or Smoking Reduction or Smoking Cessation or Tobacco-Use-Cessation or Stop* Smoking or Give* up Smoking or Quit* Smoking or Tobacco Consumption or Tobacco Chewing or Tobacco Smoke Inhalation or vaping or Vape or electronic cigarette* or e-cigarette or Smokeless Tobacco-Cessation or smoking Behaviour* or Smoking Habit* or Nicotine-Use or Smoke* or Non-smoke*OR Abstaining smoker* or abstinent smoker or ex-smoker* or former smoker* or past smoker* or smoking abstainer* or smoking regulation* or current smoker* or non-smoker or bidi smoking or hookah or dipping tobacco or naswar or smoking device* or smoking pipe* or tobacco snuff or vapers or smoking abstination or Smoking dishabituation or nicotine abstination or nicotine abstinence or nicotine withdrawal or tobacco abstinence or nicotine cessation or nicotine abstention).tw.

3 exp spine fusion/

4 (Spin*fusion* or spin* interbody fusion* or spondylosyndesis or Spin* fusion* implant* or Spondylosis or dorsal vertebral fusion* or dorsal spin* fusion* or spin* interbody fusion* or spondylodesis or vertebral fusion* or spin* arthrodesis or spin* fusion surger* or spin* spondylosis surger* or anterior spin* fusion* or posterior interbody fusion* or posterior spin* fusion* or posterior lumbar interbody fusion* or lumbar interbody fusion* or ALIF or anterior lumbar interbody fusion* or PLIF or transforaminal interbody fusion* or TLIF or lateral lumbar interbody fusion* or LLIF or direct lateral interbody fusion* or DLIF or Extreme Lateral interbody fusion* or XLIF or Thoracolumbar fusion* or multi-level anterior cervical discectomy or lumbar interbody fusion* or minimally invasive transforaminal interbody fusion* or Spondylosyndeses or Spondylodeses or posterolateral gutter fusion* or Multi-level spinal fusion* or instrumented spinal fusion surger* or lumbar fusion or anterior interbody fusion* or anterior vertebra fusion* or anterior vertebral fusion* or cervical spin* anterior interbody fusion* or spin* anterior fusion* or posterolateral spin* fusion*).tw.

5 1 or 2

6 3 or 4

7 5 and 6

8 limit 7 to human

#1218 hits in August 2024.

***COCHRANE SEARCH STRATEGY***

**Date Run:** August, 2024

ID Search Hits

#1 MeSH descriptor: [Smoking] explode all trees

#2 (Tobacco use OR pipe smok* OR smoking reduction OR smoking cessation OR Tobacco cessation OR Tobacco smoke OR Cigar smoke* OR cigarette smoke*OR smoking reduction R smoking cessation OR tobacco use cessation OR stopping smoking OR giv* up smoking OR quit* smoking OR tobacco consumption OR tobacco consumption OR tobacco chewing OR Tobacco smoke inhalation OR vaping OR vape OR electronic cigarette* OR smokeless tobacco cessation OR smoking behaviour* OR smoking habit* OR nicotine use OR smoke OR Non smoke* OR abstaining smoker* OR abstinent smoker* OR former smoker* OR past smoker* OR smoking abstainer* OR smoking regulation* OR current smoker* OR non-smoker OR bidi smoking OR hookah OR dipping tobacco OR naswar OR smoking device* OR Smoking pipes OR tobacco snuff OR vapers OR E NEXT cigarette* OR ex smoker* OR smoking abstination OR Smoking dishabituation OR nicotine abstination OR nicotine abstinence OR nicotine withdrawal OR Tobacco abstinence OR nicotine cessation OR nicotine abstention):ti,ab,kw

#3 MeSH descriptor: [Spinal Fusion] explode all trees

#4 (Spin* fusion* OR spin* interbody fusion* OR Spin* fusion* implant* OR Spondylosis OR dorsal spin* fusion* OR spin* fusion* OR vertebral fusion* OR spin* arthrodesis OR spin* fusion* surger* OR anterior spin* fusion* OR posterior interbody fusion* OR posterior spin* fusion* OR posterior lumbar interbody fusion* OR lumbar interbody fusion* OR ALIF OR anterior lumbar interbody fusion* OR PLIF OR transforaminal interbody fusion* OR TLIF OR lateral lumbar interbody fusion* OR LLIF OR direct lateral interbody fusion* OR DLIF OR Extreme Lateral interbody fusion* OR XLIF OR Thoracolumbar fusion* OR multi-level anterior cervical discectomy OR lumbar interbody fusion* OR minimally invasive transforaminal interbody fusion* OR posterolateral gutter fusion* OR Multi-level spinal fusion* OR lumbar fusion* OR lumbar fusion* OR anterior interbody fusion* OR anterior vertebra fusion* OR anterior vertebral fusion* OR cervical spin* anterior interbody fusion* OR spin* anterior fusion* OR posterolateral spin* fusion*):ti,ab,kw

#5 #1 OR #2

#6 #3 OR #4

#7 #5 AND #6

74 hits on August, 2024.
