# Supplementary material for: The association between smoking and clinical outcomes among spondylodesis patients: A systematic review and meta-analysis
Source: PLoS One. 2026 Jan 13;21(1):e0337799. doi: 10.1371/journal.pone.0337799 (PMC12799005; doi:10.1371/journal.pone.0337799)
Supplement: S2 Appendix — Description of method used for assessment of risk of bias in cohort studies. (DOCX) [file pone.0337799.s002.docx]

**Appendix S2a.** Coding manual for cohort studies – Newcastle-Ottawa Scale

*Selection*

1. Representation of the exposed cohort

Truly representative or somewhat representative of the community – awarded one star.

1. Selection of the non-exposed cohort

From the same community as the exposed cohort – awarded one star.

1. Ascertainment of exposure

Secure record or structured interview – awarded one star.

1. Demonstration that outcome was not present at the start of the study.

Yes – awarded one star.

*Comparability*

1. Comparability of cohorts based on design or analysis.
   1. Study comparable at baseline – awarded one star.
   2. Study controls for additional factors such as BMI, age, sex, osteoporosis, in regression – awarded two stars.

*Outcome*

1. Assessment of outcome
   1. Independent blind assessment – award one star.
   2. Record linkage – award one star.
2. Was follow-up long enough?
   1. Yes – award one star.
3. Adequacy of follow-up of cohorts
   1. Complete follow up i.e., all subjects accounted for – award one star.
   2. Subjects lost to follow-up are unlikely to introduce bias – award one star.
