# Supplementary material for: The association between smoking and clinical outcomes among spondylodesis patients: A systematic review and meta-analysis
Source: PLoS One. 2026 Jan 13;21(1):e0337799. doi: 10.1371/journal.pone.0337799 (PMC12799005; doi:10.1371/journal.pone.0337799)
Supplement: S3 Appendix — Overview of the study quality assessment for included cohort studies. (DOCX) [file pone.0337799.s003.docx]

**Appendix S2b.** Study quality assessment using Newcastle-Ottawa Scale for cohorts – results.

| **Author name (year)** | **Selection 1** | **Selection 2** | **Selection 3** | **Selection 4** | **Comparability** | **Outcome 1** | **Outcome 2** | **Outcome 3** | **Total (max = 9)** |
| --- | --- | --- | --- | --- | --- | --- | --- | --- | --- |
| Brown (1985) | 1 | 1 | 1 | 1 | 1 | 0 | 0 | 1 | 6  Fair |
| Bydon (2014) | 1 | 1 | 1 | 1 | 1 | 1 | 0 | 1 | 7  Good |
| Cerier (2019) | 1 | 1 | 0 | 1 | 1 | 1 | 1 | 0 | 6  Fair |
| Eubanks (2011) | 1 | 1 | 0 | 1 | 0 | 0 | 1 | 1 | 5  Fair |
| Gatot (2022) | 1 | 1 | 1 | 1 | 2 | 1 | 1 | 1 | 9  Good |
| Glassman (2000) | 1 | 1 | 1 | 1 | 0 | 1 | 1 | 1 | 7  Good |
| Goyal (2021) | 1 | 1 | 1 | 1 | 1 | 1 | 1 | 1 | 8  Good |
| Hermann (2016) | 1 | 1 | 1 | 1 | 0 | 0 | 1 | 0 | 5  Fair |
| Hilibrand (2001) | 1 | 1 | 1 | 1 | 0 | 0 | 1 | 1 | 6  Fair |
| Jazini (2018) | 1 | 1 | 1 | 1 | 0 | 0 | 1 | 1 | 6  Fair |
| Joswig (2016) | 1 | 1 | 0 | 1 | 2 | 1 | 1 | 1 | 8  Good |
| Kruk (2024) | 1 | 1 | 1 | 1 | 0 | 1 | 1 | 1 | 7  Good |
| Kuo (2020) | 1 | 1 | 0 | 1 | 0 | 1 | 1 | 1 | 6  Fair |
| Kusin (2015) | 1 | 1 | 1 | 1 | 2 | 1 | 1 | 1 | 9  Good |
| Lau (2014) | 1 | 1 | 1 | 1 | 2 | 0 | 1 | 1 | 8  Good |
| Luca (2011) | 1 | 1 | 1 | 1 | 0 | 1 | 1 | 1 | 7  Good |
| Luszczyk (2013) | 0 | 1 | 0 | 1 | 0 | 1 | 1 | 1 | 5  Fair |
| Macki (2017) | 0 | 1 | 1 | 1 | 2 | 0 | 1 | 1 | 7  Good |
| Mangan (2021) | 1 | 1 | 1 | 1 | 2 | 1 | 1 | 1 | 9  Good |
| Nagoshi (2020) | 1 | 1 | 1 | 1 | 2 | 1 | 1 | 1 | 9  Good |
| Patel (2019) | 1 | 1 | 0 | 1 | 0 | 1 | 1 | 1 | 6  Fair |
| Phan (2018) | 1 | 1 | 0 | 1 | 0 | 1 | 1 | 1 | 6  Fair |
| Senker (2021) | 0 | 0 | 0 | 1 | 0 | 0 | 1 | 0 | 2  Poor |
| Toci (2022) | 1 | 1 | 1 | 1 | 2 | 1 | 1 | 1 | 9  Good |
| Tu (2019) | 1 | 1 | 0 | 1 | 0 | 1 | 1 | 1 | 6  Fair |
| Wang (2021) | 1 | 1 | 1 | 1 | 2 | 1 | 1 | 1 | 9  Good |
| Wen-Shen (2020) | 1 | 1 | 1 | 1 | 0 | 1 | 1 | 1 | 7  Good |

One score is equivalent to 1 star awarded per checklist item.

Note: Rating score is categorized as follows: 0-3 points = “poor quality”; 4-6 points = “fair quality”; and 7-9 points = “good quality”.
