# Supplementary material for: The association between smoking and clinical outcomes among spondylodesis patients: A systematic review and meta-analysis
Source: PLoS One. 2026 Jan 13;21(1):e0337799. doi: 10.1371/journal.pone.0337799 (PMC12799005; doi:10.1371/journal.pone.0337799)
Supplement: S4 Appendix — Description of the method used for assessment of risk of bias in case series. (DOCX) [file pone.0337799.s004.docx]

**Appendix S3a.** Joanna Briggs Institute Critical Appraisal Checklist for Case Series (2020) – Questions

Q1. Were there clear criteria for inclusion in the case series?

Q2. Was the condition measured in a standard, reliable way for all participants included in the case series?

Q3. Were valid methods used for identification of the condition for all participants included in the case series?

Q4. Did the case series have consecutive inclusion of participants?

Q5. Did the case series have consecutive inclusion of participants?

Q6. Was there clear reporting of the demographics of the participants in the study?

Q7. Was there clear reporting of clinical information of the participants?

Q8. Were the outcomes or follow up results of cases clearly reported?

Q9. Was there clear reporting of the presenting site(s)/clinic(s) demographic information?

Q10. Was statistical analysis appropriate?
