# Supplementary material for: The association between smoking and clinical outcomes among spondylodesis patients: A systematic review and meta-analysis
Source: PLoS One. 2026 Jan 13;21(1):e0337799. doi: 10.1371/journal.pone.0337799 (PMC12799005; doi:10.1371/journal.pone.0337799)
Supplement: S5 Appendix — Overview of study quality assessment for included case series. (DOCX) [file pone.0337799.s005.docx]

**Appendix S3b.** Joanna Briggs Institute Critical Appraisal Checklist for Case Series (2020) – Results

| **Author name (Year)** | **Design** | **Answer based on appropriate JBI appraisal** | | | | | | | | | | **Quality score** |
| --- | --- | --- | --- | --- | --- | --- | --- | --- | --- | --- | --- | --- |
|  |  | **1** | **2** | **3** | **4** | **5** | **6** | **7** | **8** | **9** | **10** |  |
| Bertagnoli (2006) | Y | Y | Y | Y | Y | N | Y | Y | N | U | U | 7  Moderate |
| Bose (2001) | N | Y | Y | N | N | Y | Y | Y | N | N | N | 5  Moderate |

Abbreviations: JBI = Joanna Briggs Institute, Y = yes, N= no, U = unclear Note: rating score is from 1 (lowest) to 10 (highest); quality score was categorized into three groups: Low = 1-4, Moderate = 5-7, Good = 8-10
