# Supplementary material for: The association between smoking and clinical outcomes among spondylodesis patients: A systematic review and meta-analysis
Source: PLoS One. 2026 Jan 13;21(1):e0337799. doi: 10.1371/journal.pone.0337799 (PMC12799005; doi:10.1371/journal.pone.0337799)
Supplement: S7 Appendix — (DOCX) [file pone.0337799.s007.docx]

**Appendix S5.** Forest plot depicting event rate of pseudoarthrosis in smokers and non-smokers.

Forest plot showing the event rate (95% confidence interval (CI)) in smokers and non-smokers; the horizontal line segments represent the individual study’s CI, the square represents the event rate of each study, and the center of the diamond represents the summary effect for the pooled studies. The size of the solid square is proportional to the weight of the study.
