# Supplementary material for: The association between smoking and clinical outcomes among spondylodesis patients: A systematic review and meta-analysis
Source: PLoS One. 2026 Jan 13;21(1):e0337799. doi: 10.1371/journal.pone.0337799 (PMC12799005; doi:10.1371/journal.pone.0337799)
Supplement: S1 Table — (DOCX) [file pone.0337799.s014.docx]

**Supplementary table S1.** Comparison of the difference between mean NDI scores along with the relative mean difference for smokers and non-smokers across the original studies.

|  | **Smokers** | | | | **Non-smokers** | | | |
| --- | --- | --- | --- | --- | --- | --- | --- | --- |
| **First author, publication year** | **Pre-operative (mean ± SD)** | **Post-operative (mean ± SD)** | **Pre minus post operative (mean ± SD)^** | **Relative difference from baseline (mean ± SD)** | **Pre-operative (mean ± SD)** | **Post-operative (mean ± SD)** | **Pre minus post operative (mean ± SD)^** | **Relative difference from baseline (mean ± SD)** |
| Cerier E, 2019 | 62.8 ± 12.7 | 45.5 ± 17.7 | 17.4 ± 31.9 | 27.7 **±** 0.5 | 45.9 ± 15.3 | 15.9 ± 12.1 | 30.0 ± 27.4 | **65.4 ± 0.3** |
| Patel D, 2019 | 44.9 ± 20.6 | 14.5 ± 4.5 | 18.8 ± 27.8 | 41.9 **±** 0.2 | 40.2 ± 19.5 | 13.0 ± 3.6 | 17.6 ± 18.2 | **43.8 ± 0.1** |
| Tu T, 2019 | 15.3 ± 7.3 | 5.2 ± 5.1 | 10.1 ± 12.6 | **66.0 ± 0.4** | 15.8 ± 11.9 | 7.8 ± 7.0 | 8.0 ± 19.5 | 50.6 ± 1.2 |
| *Mangan J. 2021 | 46.5 ± 16.9 | 30.0 ± 22.9 | 16.5 ± 42.8 | 35.5 ± 0.5 | 41.7 ± 24.7 | 22.8 ± 29.9 | 18.9 ± 54.9 | **45.3 ± 0.8** |
| Wang H, 2021 | 30.5 ± 3.6 | 8.5 ± 2.6 | 22.0 ± 6.4 | 72.1 ± 0.1 | 31.1 ± 3.9 | 7.9 ± 2.7 | 23.2 ± 6.8 | **74.6 ± 0.09** |
| *Toci G, 2022 | 41.2 ± 17.6 | 35.7 ± 23.9 | 5.5 ± 21.0 | 13.3 ± 0.7 | 38.4 ± 21.2 | 31.3 ± 21.9 | 7.1 ± 19.8 | **18.5 ± 0.7** |

Abbreviations: NDI = Neck disability index, SD = standard deviation.
Bold indicates more favorable outcomes observed in one group or the other. Five out of six studies showed more favorable outcomes in the non-smokers than in smokers.
*Indicate studies that stratified non-smokers into former smokers and never smokers
