# Supplementary material for: The association between smoking and clinical outcomes among spondylodesis patients: A systematic review and meta-analysis
Source: PLoS One. 2026 Jan 13;21(1):e0337799. doi: 10.1371/journal.pone.0337799 (PMC12799005; doi:10.1371/journal.pone.0337799)
Supplement: S2 Table — (DOCX) [file pone.0337799.s015.docx]

**Supplementary table S2.** Comparison of the difference between mean NDI scores along with the relative mean difference for smokers, former smokers, and never smokers across different studies.

|  | **Smokers** | | | | **Former Smokers** | | | | **Never Smokers** | | | |
| --- | --- | --- | --- | --- | --- | --- | --- | --- | --- | --- | --- | --- |
| **First author, publication year** | **Pre-operative (mean ± SD)** | **Post-operative (mean ± SD)** | **Pre minus post operative (mean ± SD)** | **Relative difference from baseline (mean ± SD)** | **Pre-operative (mean ± SD)** | **Post-operative (mean ± SD)** | **Pre minus post operative (mean ± SD)** | **Relative difference from baseline (mean ± SD)** | **Pre-operative (mean ± SD)** | **Post-operative (mean ± SD)** | **Pre minus post operative (mean ± SD)** | **Relative difference from baseline (mean ± SD)** |
| Mangan J, 2021 | 46.5 ± 16.9 | 30 ± 22.9 | 16.5 ± 28.5 | 35.5 ± 0.5 | 45.2 ± 19.7 | 28.4 ± 22.7 | 16.8 ± 30.1 | 37.2 ± 0.6 | 40.1 ± 20.4 | 23.4 ± 22.3 | 16.7 ± 30.2 | 41.6 ± 0.6 |
| Toci G, 2022 | 41.2 ± 17.6 | 35.7 ± 23.9 | 5.3 ± 21.8 | 12.9 ± 0.7 | 38.0 ± 21.8 | 34.9 ± 23.9 | 3.1 ± 17.6 | 8.2 ± 0.8 | 38.6 ± 21.0 | 29.5 ± 20.7 | 9.1 ± 20.9 | 23.6 ± 0.7 |

Bold indicates more favorable outcomes observed in one group or the other. Five out of six studies showed more favorable outcomes in the non-smokers than in smokers.
