# Supplementary material for: The association between smoking and clinical outcomes among spondylodesis patients: A systematic review and meta-analysis
Source: PLoS One. 2026 Jan 13;21(1):e0337799. doi: 10.1371/journal.pone.0337799 (PMC12799005; doi:10.1371/journal.pone.0337799)
Supplement: S3 Table — (DOCX) [file pone.0337799.s016.docx]

**Supplementary table S3.** Comparison of the difference between mean ODI scores along with the relative mean difference for smokers and non-smokers across the original studies.

|  | **Smokers** | | | | **Non-smokers** | | | |
| --- | --- | --- | --- | --- | --- | --- | --- | --- |
| **First author, publication year** | **Pre-operative (mean ± SD)** | **Post-operative (mean ± SD)** | **Pre minus post operative (mean ± SD)** | **Relative difference from baseline (mean ± SD)** | **Pre-operative (mean ± SD)** | **Post-operative (mean ± SD)** | **Pre minus post operative (mean ± SD)** | **Relative difference from baseline (mean ± SD)** |
| Bertagnoli R, 2006 | 55.0 ± N.G. | 28.0 ± N.G. | 27.0 ± N.G. | **49.1 ± N.G.** | 52.0 ± N.G. | 32.0 ± N.G. | 20.0 ± N.G. | 38.5 ± N.G. |
| Hermann P, 2016 | 70.0 ± 12.0 | 28.0 ± 22.0 | 42.0 ± 35.4 | 60.0 ± 0.3 | 68.0 ± 14.0 | 26.0 ± 21.0 | 45.0 ± 35.7 | **66.2 ± 0.3** |
| Phan K, 2017 | 65.5 ± 19.6 | 32.6 ± 21.9 | 32.9 ± 21.7 | 50.2 ± 0.4 | 56.4 ± 22.6 | 27.9 ± 18.8 | 31.5 ± 25.0 | **55.9 ± 0.4** |
| *Jazini E, 2018 | 58.3 ± 15.3 | 45.5 ± 22.3 | 12.8 ± 38.2 | 22.0 ± 0.4 | 50.4 ± 15.4 | 31.1 ± 20.7 | 12.8 ± 36.3 | **31.4 ± 0.5** |
| *Goyal D, 2021 | 44.1 ± 6.8 | 25.5 ± 10.6 | 17.1 ± 28.3 | 38.8 ± 0.3 | 40.8 ± 2.3 | 20.3 ± 2.3 | 20.8 ± 38.0 | **51.0 ± 0.1** |
| Gatot C, 2022 | 44.5 ± 19.0 | 13.4 ± 20.8 | 32.1 ± 29.8 | 72.1 ± 0.5 | 47.2 ± 17.4 | 10.5 ± 11.1 | 36.7 ± 29.2 | **77.8 ± 0.3** |

Abbreviations: ODI = Oswestry disability index, SD = standard deviation, N.G. = not given.
Bold indicates more favorable outcomes observed in one group or the other. Five out of six studies showed more favorable outcomes in the non-smokers than in smokers.
*Indicate studies that stratified non-smokers into former smokers and never smokers
