# Supplementary material for: The association between smoking and clinical outcomes among spondylodesis patients: A systematic review and meta-analysis
Source: PLoS One. 2026 Jan 13;21(1):e0337799. doi: 10.1371/journal.pone.0337799 (PMC12799005; doi:10.1371/journal.pone.0337799)
Supplement: S5 Table — (DOCX) [file pone.0337799.s018.docx]

**Supplementary Table S5.** Comparison of the difference between VAS neck pain scores along with the relative mean difference for smokers and non-smokers across different studies.

|  | **Smokers** | | | | **Non-smokers** | | | |
| --- | --- | --- | --- | --- | --- | --- | --- | --- |
| **First author, publication year** | **Pre-operative (mean ± SD)** | **Post-operative (mean ± SD)** | **Pre minus post operative (mean ± SD)** | **Relative difference from baseline (mean ± SD)** | **Pre-operative (mean ± SD)** | **Post-operative (mean ± SD)** | **Pre minus post operative (mean ± SD)** | **Relative difference from baseline (mean ± SD)** |
| Tu T, 2019 | 4.5 ± 3.2 | 2.2 ± 2.4 | 2.3 ± 5.6 | **51.1 ± 0.6** | 4.7 ± 3.4 | 2.6 ± 2.7 | 2.1 ± 6.1 | 44.7 ± 0.7 |
| Patel D, 2019 | 7.2 ± 2.1 | 3.2 ± 1.0 | 4.0 ± 3.9 | **55.6 ± 0.2** | 5.9 ± 2.6 | 2.9 ± 0.4 | 3.0 ± 3.0 | 50.8 ± 0.2 |
| Nagoshi N, 2020 | 4.0 ± 2.8 | 2.3 ± 1.3 | 1.7 ± 3.1 | 42.5 ± 0.5 | 3.4 ± 2.9 | 2.0 ± 0.8 | 1.4 ± 2.8 | 41.2 ± 0.6 |
| *Wang H, 2021 | 6.0 ± 0.8 | 1.5 ± 0.5 | 4.5 ± 1.3 | 75.0 ± 0.1 | 5.8 ± 1.1 | 1.5 ± 0.6 | 4.3 ± 1.8 | 74.1 ± 0.1 |
| *Mangan J, 2021 | 6.4 ± 2.7 | 3.6 ±3.2 | 2.8 ± 5.9 | 43.8 ± 0.6 | 5.5 ± 8.0 | 3.1 ±5.3 | 2.4 ± 13.5 | 43.6 ± 1.3 |
| *Toci G, 2022 | 5.8 ± 3.0 | 2.8 ± 2.8 | 3.0 ± 9.1 | **51.7 ± 0.5** | 5.1 ± 3.3 | 2.9 ± 2.8 | 2.2 ± 6.1 | 43.1 ± 0.7 |

Abbreviations: VAS = visual analogue scale, SD = standard deviation.
Bold indicates more favorable outcomes observed in one group or the other. Five out of six studies showed more favorable outcomes in the non-smokers than in smokers.
*Indicate studies that stratified non-smokers into former smokers and never smokers.
