# Supplementary material for: The association between smoking and clinical outcomes among spondylodesis patients: A systematic review and meta-analysis
Source: PLoS One. 2026 Jan 13;21(1):e0337799. doi: 10.1371/journal.pone.0337799 (PMC12799005; doi:10.1371/journal.pone.0337799)
Supplement: S7 Table — (DOCX) [file pone.0337799.s020.docx]

**Supplementary Table S7.** Comparison of the difference between mean VAS arm pain scores along with the relative mean difference for smokers and non-smokers across different studies.

|  | **Smokers** | | | | **Non-smokers** | | | |
| --- | --- | --- | --- | --- | --- | --- | --- | --- |
| **First author, publication year** | **Pre-operative (mean ± SD)** | **Post-operative**  **(mean ± SD)** | **Pre minus post operative (mean ± SD)** | **Relative difference from baseline (mean ± SD)** | **Pre-operative (mean ± SD)** | **Post-operative (mean ± SD)** | **Pre minus post operative (mean ± SD)** | **Relative difference from baseline (mean ± SD)** |
| Patel D, 2019 | 7.1 ± 1.5 | 3.7 ± 1.1 | 3.4 ± 3.8 | 47.9 ± 0.2 | 5.7 ± 2.8 | 2.7 ± 0.5 | 3.0 ± 3.3 | **52.6 ± 0.2** |
| Tu T, 2019 | 5.1 ± 3.5 | 1.6 ± 2.6 | 3.5 ± 6.1 | **68.6 ± 0.6** | 4.3 ± 3.4 | 2.1 ± 2.5 | 2.2 ± 6.0 | 51.2 ± 0.7 |
| *Mangan J, 2021 | 5.6 ± 3.2 | 2.7 ± 2.7 | 2.9 ± 5.8 | **51.8 ± 0.6** | 5.1 ± 8.0 | 2.8 ± 5.3 | 2.3 ± 13.5 | 45.1 ± 1.4 |
| *Wang H, 2021 | 2.9 ± 1.0 | 1.6 ± 0.7 | 1.3 ± 1.7 | 44.8 ± 0.3 | 5.6 ± 1.3 | 1.6 ± 1.0 | 4.0 ± 2.3 | **71.4 ±** **0.2** |
| *Toci G, 2022 | 6.4 ± 3.1 | 2.9 ± 3.0 | 3.5 ± 6.1 | **54.7 ± 0.5** | 4.9 ± 3.1 | 2.3 ± 2.8 | 2.6 ± 5.9 | 53.1 ± 0.6 |

Abbreviations: VAS = visual analogue scale, SD = standard deviation
Bold indicates more favorable outcomes observed in one group or the other. Five out of six studies showed more favorable outcomes in the non-smokers than in smokers. *Indicate studies that stratified non-smokers into former smokers and never smokers
