# Supplementary material for: The association between smoking and clinical outcomes among spondylodesis patients: A systematic review and meta-analysis
Source: PLoS One. 2026 Jan 13;21(1):e0337799. doi: 10.1371/journal.pone.0337799 (PMC12799005; doi:10.1371/journal.pone.0337799)
Supplement: S8 Table — (DOCX) [file pone.0337799.s021.docx]

**Supplementary table S8.** Comparison of the difference between mean VAS arm pain scores along with the relative mean difference for smokers, former smokers, and never smokers across different studies.

|  | **Smokers** | | | | **Former Smokers** | | | | **Never Smokers** | | | |
| --- | --- | --- | --- | --- | --- | --- | --- | --- | --- | --- | --- | --- |
| **First author, publication year** | **Pre-operative (mean ± SD)** | **Post-operative (mean ± SD)** | **Pre minus post operative (mean ± SD)** | **Relative difference from baseline (mean ± SD)** | **Pre-operative (mean ± SD)** | **Pre minus post operative (mean ± SD)** | **Relative difference from baseline (mean ± SD)** | **Relative difference (mean ± SD)** | **Pre-operative (mean ± SD)** | **Post-operative (mean ± SD)** | **Pre minus post operative (mean ± SD)** | **Relative difference from baseline (mean ± SD)** |
| Mangan J, 2021 | 5.6 ± 3.2 | 2.7 ± 2.7 | 2.9 ± 2.7 | 51.8 ± 0.6 | 6.0 ± 2.8 | 3.2 ± 3.0 | 2.8 ± 5.4 | 46.7 ± 0.6 | 4.7 ± 3.5 | 2.6 ± 3.2 | 2.1 ± 1.8 | 44.7 ± 0.8 |
| Wang H, 2021 | 5.9 ± 1.0 | 1.6 ± 0.7 | 4.3 ± 1.2 | 72.9 ± 0.1 | 5.4 ± 3.0 | 1.9 ± 2.4 | 3.5 ± 3.8 | 64.8 ± 0.5 | 5.7 ± 1.2 | 1.6 ± 0.9 | 4.1 ± 1.5 | 71.9 ± 0.2 |
| Toci G, 2022 | 6.4 ± 3.1 | 2.9 ± 3.0 | 3.5 ± 4.3 | 54.7 ± 0.5 | 5.4 ± 1.4 | 1.4 ± 1.1 | 4.0 ± 1.8 | 74.1 ± 0.2 | 4.8 ± 3.2 | 2.5 ± 2.9 | 2.3 ± 4.3 | 47.9 ± 0.7 |

Bold indicates more favorable outcomes observed in one group or the other. Five out of six studies showed more favorable outcomes in the non-smokers than in smokers.
