# Supplementary material for: The association between smoking and clinical outcomes among spondylodesis patients: A systematic review and meta-analysis
Source: PLoS One. 2026 Jan 13;21(1):e0337799. doi: 10.1371/journal.pone.0337799 (PMC12799005; doi:10.1371/journal.pone.0337799)
Supplement: S9 Table — (DOCX) [file pone.0337799.s022.docx]

**Supplementary Table S9.** Comparison of the difference between mean VAS back pain scores along with the relative mean difference for smokers and non-smokers across different studies.

|  | **Smokers** | | | | **Non-smokers** | | | |
| --- | --- | --- | --- | --- | --- | --- | --- | --- |
| **First author, publication year** | **Pre-operative (mean ± SD)** | **Post-operative (mean ± SD)** | **Pre minus post operative (mean ± SD)** | **Relative difference from baseline (mean ± SD)** | **Pre-operative (mean ± SD)** | **Post-operative (mean ± SD)** | **Pre minus post operative (mean ± SD)** | **Relative difference from baseline (mean ± SD)** |
| Bertagnoli R, 2006 | 7.5 ± N.G. | 4.5 ± N.G. | 3.0 ± N.G. | 40.0 ± N.G. | 7.5 ± N.G. | 3.8 ± N.G. | 3.7 ± N.G. | **49.3 ± N.G.** |
| Hermann P, 2016 | 8.2 ± 2.2 | 3.2 ± 2.4 | 5.0 ± 4.6 | **61.0 ± 0.3** | 8.1 ± 1.8 | 3.6 ± 2.5 | 4.5 ± 4.4 | 55.6 ± 0.3 |
| *Jazini E, 2018 | 7.8 ± 1.7 | 5.3 ± 2.9 | 2.5 ± 4.8 | 32.1 ± 0.4 | 7.0 ± 2.1 | 3.9 ± 2.8 | 3.1 ± 5.0 | **44.3 ± 0.4** |
| *Goyal D, 2021 | 6.2 ± 3.3 | 3.2 ± 2.9 | 3.0 ± 6.2 | 48.4 ± 0.5 | 5.8 ± 5.9 | 2.7 ± 6.3 | 3.1 ± 12.3 | **53.4 ± 1.2** |
| Gatot C, 2022 | 6.0 ± 2.5 | 1.3 ± 2.6 | 4.7 ± 5.1 | 78.3 ± 0.4 | 6.1 ± 2.8 | 0.7 ± 1.9 | 5.4 ± 4.9 | **88.5 ± 0.3** |

Abbreviations: VAS = visual analogue scale, SD = standard deviation, N.G. = not given
Bold indicates more favorable outcomes observed in one group or the other. Five out of six studies showed more favorable outcomes in the non-smokers than in smokers. *Indicate studies that stratified non-smokers into former smokers and never smokers
