# Supplementary material for: The association between smoking and clinical outcomes among spondylodesis patients: A systematic review and meta-analysis
Source: PLoS One. 2026 Jan 13;21(1):e0337799. doi: 10.1371/journal.pone.0337799 (PMC12799005; doi:10.1371/journal.pone.0337799)
Supplement: S10 Table — (DOCX) [file pone.0337799.s023.docx]

**Supplementary table S10.** Comparison of the difference between mean VAS back pain scores along with the relative mean difference for smokers, former smokers, and never smokers across different studies.

|  | **Smokers** | | | | **Former Smokers** | | | | **Never Smokers** | | | |
| --- | --- | --- | --- | --- | --- | --- | --- | --- | --- | --- | --- | --- |
| **First author, publication year** | **Pre-operative (mean ± SD)** | **Post-operative (mean ± SD)** | **Pre minus post operative (mean ± SD)** | **Relative difference from baseline (mean ± SD)** | **Pre-operative (mean ± SD)** | **Post-operative (mean ± SD)** | **Pre minus post operative (mean ± SD)** | **Relative difference from baseline (mean ± SD)** | **Pre-operative (mean ± SD)** | **Post-operative (mean ± SD)** | **Pre minus post operative (mean ± SD)** | **Relative difference from baseline (mean ± SD)** |
| Jazini E, 2018 | 7.8 ± 1.7 | 5.3 ± 2.9 | 2.5 ± 3.4 | 32.1 ± 0.4 | 7.1 ± 2.1 | 4.4 ± 2.9 | 2.7 ± 3.6 | 38.0 ± 0.3 | 7.0 ± 2.2 | 3.7 ± 2.8 | 3.3 ± 3.6 | 47.1 ± 0.6 |
| Goyal D, 2021 | 6.2 ± 3.3 | 3.2 ± 2.9 | 3.0 ± 4.4 | 48.4 ± 0.5 | 5.9 ± 3.2 | 3.1 ± 2.7 | 2.8 ± 4.2 | 47.5 ± 0.5 | 5.7 ± 3.0 | 2.5 ± 2.7 | 3.2 ± 4.0 | 56.1 ± 0.5 |

Bold indicates more favorable outcomes observed in one group or the other. Five out of six studies showed more favorable outcomes in the non-smokers than in smokers.
