# Supplementary material for: The association between smoking and clinical outcomes among spondylodesis patients: A systematic review and meta-analysis
Source: PLoS One. 2026 Jan 13;21(1):e0337799. doi: 10.1371/journal.pone.0337799 (PMC12799005; doi:10.1371/journal.pone.0337799)
Supplement: S11 Table — (DOCX) [file pone.0337799.s024.docx]

**Supplementary table S11.** Comparison of the difference between mean VAS leg pain scores along with the relative mean difference across different studies.

|  | **Smokers** | | | | **Non-smokers** | | | |
| --- | --- | --- | --- | --- | --- | --- | --- | --- |
| **First author, publication year** | **Pre-operative smokers (mean ± SD)** | **Post-operative smokers (mean ± SD)** | **Pre minus post operative (mean ± SD)** | **Relative difference from baseline (mean ± SD)** | **Pre-operative non-smokers (mean ± SD)** | **Post-operative non-smokers (mean ± SD)** | **Pre minus post operative (mean ± SD)** | **Relative difference from baseline (mean ± SD)** |
| *Jazini E, 2018 | 7.1 ± 2.4 | 4.8 ± 3.2 | 2.3 ± 5.7 | **84.3 ± 0.5** | 6.7 ± 2.4 | 3.1 ± 3.0 | 3.6 ± 5.4 | 53.7 ± 0.5 |
| *Goyal D, 2021 | 6.4 ± 3.5 | 3.6 ± 3.5 | 2.8 ± 70.4 | 43.8 ± 0.5 | 6.0 ± 8.6 | 2.1 ± 5.4 | 3.9 ± 10.2 | **65.0 ± 1.0** |
| Gatot C, 2022 | 5.1 ± 3.5 | 0.8 ± 2.4 | 4.3 ± 6.0 | 84.3 ± 0.5 | 5.8 ± 3.3 | 0.4 ± 1.5 | 5.4 ± 5.0 | **93.1 ± 0.3** |

Abbreviations: VAS = visual analogue scale, SD = standard deviation
Bold indicates more favorable outcomes observed in one group or the other. Five out of six studies showed more favorable outcomes in the non-smokers than in smokers. *Indicate studies that stratified non-smokers into former smokers and never smokers
