# Supplementary material for: The association between smoking and clinical outcomes among spondylodesis patients: A systematic review and meta-analysis
Source: PLoS One. 2026 Jan 13;21(1):e0337799. doi: 10.1371/journal.pone.0337799 (PMC12799005; doi:10.1371/journal.pone.0337799)
Supplement: S13 Table — (DOCX) [file pone.0337799.s026.docx]

**Supplementary table S13.** Comparison of the difference between mean JOA scores along with the relative mean difference for smokers and non-smokers across different studies.

|  | **Smokers** | | | | **Non-smokers** | | | |
| --- | --- | --- | --- | --- | --- | --- | --- | --- |
| **First author, publication year** | **Pre-operative (mean ± SD)** | **Post-operative (mean ± SD)** | **Post minus pre operative (mean ± SD)** | **Relative difference from baseline (mean ± SD)** | **Pre-operative (mean ± SD)** | **Post-operative (mean ± SD)** | **Post minus pre operative (mean ± SD)** | **Relative difference from baseline (mean ± SD)** |
| Tu T, 2019 | 12.5 ± 1.6 | 14.8 ± 2.0 | 2.3 ± 3.6 | **18.4 ± 0.2** | 13.1 ± 3.2 | 14.7 ± 2.3 | 1.6 ± 5.6 | 12.2 ± 0.3 |
| Nagoshi N, 2020 | 11.0 ± 2.4 | 7.8 ± 1.2 | 3.2 ± 2.1 | **29.1 ± 0.2** | 11.1 ± 2.4 | 8.1 ± 1.2 | 3.0 ± 2.1 | 27.0 ± 0.2 |
| *Wang H, 2021 | 10.6 ± 1.0 | 15.6 ± 0.8 | 5.0 ± 1.9 | **47.2 ± 0.2** | 10.9 ± 1.5 | 15.8 ± 1.1 | 4.9 ± 2.6 | 45.0 ± 0.2 |
| *Toci G, 2022 | 12.2 ± 3.4 | 13.0 ± 2.4 | 0.8 ± 3.5 | **6.6 ± 0.4** | 14.0 ± 2.9 | 14.0 ± 3.0 | 0.2 ± 3.3 | 1.4 ± 0.3 |

Abbreviations: JOA = Japanese orthopaedic association, SD = standard deviation
Bold indicates more favorable outcomes observed in one group or the other. Five out of six studies showed more favorable outcomes in the non-smokers than in smokers. *Indicate studies that stratified non-smokers into former smokers and never smokers
