# Supplementary material for: The association between smoking and clinical outcomes among spondylodesis patients: A systematic review and meta-analysis
Source: PLoS One. 2026 Jan 13;21(1):e0337799. doi: 10.1371/journal.pone.0337799 (PMC12799005; doi:10.1371/journal.pone.0337799)
Supplement: S14 Table — (DOCX) [file pone.0337799.s027.docx]

**Supplementary table S14.** Comparison of the difference between mean JOA scores along with the relative mean difference for smokers, former smokers, and never smokers across different studies.

|  | **Smokers** | | | | **Former Smokers** | | | | **Never Smokers** | | | |
| --- | --- | --- | --- | --- | --- | --- | --- | --- | --- | --- | --- | --- |
| **First author, publication year** | **Pre-operative (mean ± SD)** | **Post-operative (mean ± SD)** | **Post minus pre operative (mean ± SD)** | **Relative difference from baseline (mean ± SD)** | **Pre-operative (mean ± SD)** | **Post-operative (mean ± SD)** | **Prost minus pre operative (mean ± SD)** | **Relative difference from baseline (mean ± SD)** | **Pre-operative (mean ± SD)** | **Post-operative (mean ± SD)** | **Post minus pre operative (mean ± SD)** | **Relative difference from baseline (mean ± SD)** |
| Wang H, 2021 | 10.6 ± 1.0 | 15.6 ± 0.8 | 5.0 ± 1.3 | 47.2 ± 0.2 | 11.1 ± 1.6 | 15.8 ± 1.2 | 4.7 ± 0.3 | 42.3 ± 0.2 | 10.8 ± 1.4 | 15.8 ± 1.1 | 5.8 ± 1.8 | 53.7 ± 0.2 |
| Toci G, 2022 | 12.2 ± 3.4 | 13.0 ± 2.4 | 0.8 ± 3.5 | 6.6 ± 0.4 | 14.1 ± 2.8 | 14.5 ± 2.8 | 0.4 ± 2.9 | 2.8 ± 0.3 | 13.9 ± 3.0 | 14.0 ± 3.0 | 0.1 ± 3.5 | 0.7 ± 0.3 |

Bold indicates more favorable outcomes observed in one group or the other. Five out of six studies showed more favorable outcomes in the non-smokers than in smokers.
